# Alprazolam Reduces Freezing of Gait (FOG) and Improves FOG-Related Gait Deficiencies

**DOI:** 10.1155/2024/3447009

**Published:** 2024-01-09

**Authors:** Abdullah Al Jaja, Téa Sue, Margaret Prenger, Ken N. Seergobin, Jessica A. Grahn, Penny A. MacDonald

**Affiliations:** ^1^The Brain and Mind Institute, Western University, London, ON, Canada; ^2^Department of Neuroscience, Schulich School of Medicine and Dentistry, Western University, London, ON, Canada; ^3^Department of Medicine, University of Ottawa, Ottawa, ON, Canada; ^4^BrainsCAN, Western University, London, ON, Canada; ^5^Department of Psychology, Western University, London, ON, Canada; ^6^Department of Clinical Neurological Sciences, Western University, London, ON, Canada

## Abstract

**Background:**

Freezing of gait (FOG) is an intractable motor symptom in Parkinson's disease (PD) that increases fall risk and impairs the quality of life. FOG has been associated with anxiety, with experimental support for the notion that anxiety itself provokes FOG. We investigated the effect of acute anxiety reduction via alprazolam on FOG in PD.

**Methods:**

In ten patients with PD, FOG, and normal cognition, we administered 0.25 mg alprazolam in one session and placebo in another, in counterbalanced order. At each session, on separate days, patients walked on a pressure-sensitive walkway. Using Oculus Rift virtual-reality goggles, patients walked along a plank that appeared to be (a) level with the floor, in the low-anxiety condition or (b) raised high above the ground, in the high-anxiety conditions. In this way, we assessed the impacts of anxiety and alprazolam (i.e., anxiety reduction) on FOG frequency and other gait parameters.

**Results:**

FOG events appeared only in the high-anxiety conditions. Alprazolam significantly reduced subjective and objective measures of anxiety, as well as the prevalence of FOG (*p* = 0.05). Furthermore, alprazolam improved swing time (*p* < 0.05) and gait variability in all conditions, particularly during the elevated plank trials. *Interpretation*. Our results suggest that (1) anxiety induces FOG, and (2) alprazolam concomitantly reduces anxiety and FOG. Alprazolam further improved gait stability (i.e., swing time and gait variability). These findings reveal that anxiety triggers FOG in PD. Treating anxiety can reduce FOG and improve gait stability, potentially offering new therapeutic avenues for this intractable and disabling symptom in PD.

## 1. Introduction

Parkinson's disease (PD) is a neurodegenerative disorder characterized by the degeneration of dopamine-producing neurons in the substantia nigra pars compacta and thus resulting in different motor and cognitive impairments. The cardinal symptoms of PD are resting tremor, rigidity, bradykinesia, and postural instability. Additionally, PD patients can develop a debilitating symptom identified as freezing of gait (FOG), which is characterized by short, stuttering steps that slow patients' walking, often resulting in a complete stop [1–4]. FOG often results in falls and impairs quality of life for more than 50% of later-stage PD patients. Unfortunately, FOG responds poorly, if at all, to available PD treatments, including dopaminergic replacement therapy [5]. The pathophysiology underlying FOG remains unclear.

FOG has been associated with heightened levels of anxiety [5–7]. In a study directly examining the effect of anxiety on FOG, researchers contrasted the performance of PD patients with and without FOG, walking in two virtual reality (VR) environments that differed in their potential for inducing anxiety. PD patients with FOG reported higher levels of anxiety compared to those without FOG when walking through the high anxiety scenario [5]. In addition, PD patients with FOG experienced significantly more FOG episodes and spent more time frozen during the high anxiety condition compared to the low anxiety condition. Furthermore, anxiety impaired gait by decreasing velocity, shortening step length, and increasing step length- and step time-variability. This study provided direct evidence that anxiety itself induces FOG and affords a validated experimental paradigm for testing whether acute anxiety reduction would lead to improvement in FOG and/or gait parameters related to FOG.

First line pharmacological treatments for anxiety in PD, and in the general population, in fact, include selective serotonin reuptake inhibitors (SSRIs), serotonin and norepinephrine reuptake inhibitors (SNRIs), buspirone, monoamine oxidase inhibitors (MOA-I), and, for acute effects, benzodiazepines. Benzodiazepines acutely and effectively decrease the experience of anxiety and its physiological manifestations in several conditions such as generalized anxiety, social anxiety, phobias, and panic attacks. Though not a first choice for long-term use due to sedating effects, dependence, and abuse potential, a single dose of benzodiazepines can reduce anxiety quickly and reversibly. These properties of benzodiazepines make them highly favourable for initial scientific investigations of the behavioral effects of anxiety modulation on symptoms, such as FOG in the current instance. Alprazolam is one of the fastest-acting benzodiazepines, with a short half-life of 9 to 30 hours [8], that has been extensively used to alleviate subjective and objective manifestations of anxiety in many clinical conditions [8–10]. Interestingly, benzodiazepines are a first-line treatment in patients with hyperekplexia, a neurological condition in which excessive startle to tactile or acoustic stimuli leads to increased tone and freezing that bears some resemblance to FOG in PD [11]. In addition, benzodiazepines have been shown to be safe and effective in the treatment of anxiety in PD [12].

The objective of our study was to test the effects of reducing anxiety and its associated physiological manifestations, on FOG, to further our understanding of the relationship between anxiety and FOG, as well as to potentially open a new avenue for treating FOG, an intractable and disabling symptom in PD. Given its fast-acting nature and short half-life, alprazolam appeared optimal for these initial investigations. To our knowledge, no previous studies have tested the effect of anxiety alleviation, with benzodiazepines, on FOG. We hypothesized that reducing anxiety would decrease the frequency and severity of freezing in PD patients with FOG.

## 2. Materials and Methods

### 2.1. Participants

Thirteen cognitively normal PD patients with FOG participated in this study. All participants were able to ambulate without an assistive device for more than 10 meters and were not taking any medications that would contraindicate alprazolam administration. Patients had been diagnosed with PD by a neurologist, were treated with stable doses of dopamine-modulating therapy for at least three months, and were recruited through the Movement Disorders Database (London Health Sciences Centre, Canada). All participants were evaluated in two nearly identical sessions on separate days at the Brain and Mind Institute (Western University, Canada). In both sessions, patients took their usual dopaminergic therapy as prescribed. Three patients were excluded from the study due to their inability to tolerate the virtual environment or alprazolam. Therefore, statistical analyses were performed on the remaining group (*N* = 10, 4 females).

Alprazolam (0.25 mg) and placebo (i.e., cornstarch) caplets were administered one hour prior to gait testing, to every participant, in a double-blinded manner, with order of sessions counter-balanced across participants. Prior to capsule administration, patients completed the New Freezing of Gait Questionnaire (NFOG) [13] to assess FOG prevalence and severity. The Montreal Cognitive Assessment (MoCA) [14] was performed, and, for safety reasons, only patients with normal cognition were retained in the study and were provided alprazolam and placebo in separate sessions. In these sessions, they were tested using virtual-reality gait scenarios that aimed to manipulate anxiety. The Unified Parkinson's Disease Rating Scale part III (UPDRS-III) was also collected to evaluate PD motor symptom severity, approximately one hour after capsule administration in each session. UPDRS-III scoring was performed by a licensed neurologist with specialization in Movement Disorders (P.A.M).

All participants provided written informed consent. The study was conducted in accordance with the Declaration of Helsinki [15] and was approved by the Health Sciences Research Ethics Board of the Western University (REB#108579).

### 2.2. Procedures

One hour following capsule administration, we obtained a baseline measure of ambulation, in which participants walked along a 4.5 m long GAITRite mat (CIR systems Inc., Sparta, USA) without VR goggles. This allowed us to separate the effects of medication (i.e., alprazolam vs. placebo) and the virtual environment itself, on gait. Following ambulation in the baseline condition, participants wore Oculus Rift VR goggles (Meta Platforms Inc., California, USA) while walking along the GAITRite mat. The latter allowed us to collect gait variables and objectively identify episodes of FOG. The participants were tracked, and the viewpoint in the VR environment was updated in real time using three Oculus sensors (Meta Platforms Inc., California, USA).

Throughout the experiment, patients were asked to walk normally without any specific instructions about cadence, rhythm, or stride length. Following these instructions, patients were provided a short training period during which they wore the virtual reality goggles and were asked to explore their surroundings, walking in the virtual environment briefly until they felt comfortable. This helped patients acclimatize to the virtual environment and ensured that any instabilities could be detected before experimental conditions were introduced. Furthermore, gait corrections occurred prior to data recording.

**Figure 1:**
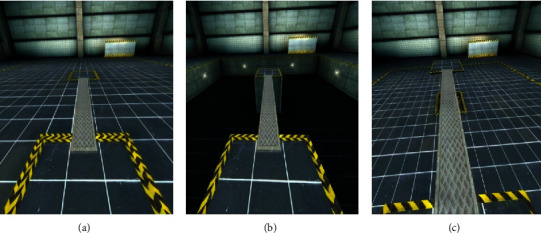
Virtual environments in which PD patients ambulated. Low anxiety (a), high anxiety-pit (b), and high anxiety-plank (c); trials were pseudorandomly presented to patients, and each block contained a combination of the three scenarios.

Three different VR environments, inspired by those used by Ehgoetz-Martens [5], were constructed using the VR software Vizard (Worldviz L.L.C., Santa Barbara, USA). The first environment was designed to evoke minimal levels of anxiety (i.e., low anxiety (LA)), in which patients walked on a virtual plank that appeared level with the floor. The second and third environments were designed to evoke higher levels of anxiety (HA). In the HA-pit condition, patients ambulated along a plank over a simulated 2.5 m deep pit. In the HA-plank condition, patients walked across a virtual plank suspended 2.5 m above ground (Figure 1). The plank was superimposed on the GAITRite mat and had identical width, length, and appearance in all conditions. In this way, the gait demands were equal across conditions, differing only in their potential to induce anxiety.

Each session was divided into 6 blocks containing five pseudorandomly assigned trials, either LA, HA-pit, or HA-plank, while ensuring that all patients experienced an equal number of trials and anxiety scenarios. We ensured that each block (5 trials) contained at least one of each anxiety conditions. A trial consisted of traversing the entire 4.5 m walkway from the starting point to the end of the mat while wearing VR goggles.

### 2.3. Anxiety Measures

Subjective anxiety was measured at several time points including prior to capsule administration and at the end of the final block using the Beck Anxiety Inventory (BAI) [16]. As the BAI asks respondents to evaluate their anxiety over the past week, a modified version of the BAI was administered with altered instructions (i.e., “How much are you bothered by the following anxiety symptoms right now?”). These instructions were tailored to the experimental setting (i.e., repeated assessment within the same experimental sessions) to measure acutely fluctuating anxiety levels. Although acute administration of the BAI has not been previously reported, the BAI has excellent face validity for panic symptoms and has been suggested for use in PD populations [17]. Additionally, objective measures of anxiety including heart rate (HR) and systolic blood pressure (BP) were obtained at the same time points as the BAI.

### 2.4. Gait Measures

Previous studies have quantified FOG in terms of the frequency of events and the duration of gait dysfunction during these episodes of FOG. In the current study, a FOG episode was identified when gait velocity (i.e., distance walked/time walked; m/s) decreased between zero and one standard deviation of the average velocity for each trial, for each participant. In keeping with previous studies [5], trials that contained FOG episodes were not included in the statistical analyses of other gait variables, to prevent variability that could obscure any other findings. Additionally, to replicate Ehgoetz Martens et al. [5] and to ensure equal representation of all VR scenarios, we analyzed only the first five trials for each LA and HA conditions throughout the study. The first five trials were chosen for analysis due to the possible fading effect of anxiety on FOG and FOG gait-related variables.

PD patients with FOG are known to have deficits in multiple spatial and temporal gait measures. For this study, gait outcome measures included step length (i.e., distance between heels of two consecutive footsteps of different feet; cm), swing time (i.e., time taken from when the toes leave the ground to when the heel strikes the ground again; s), step time (i.e., time elapsed between consecutive heel strikes of the opposite foot (e.g., right foot heel strike to the left foot heel strike); s), and cadence (i.e., steps per minute). We also measured gait variability as a percentage of the coefficient of variability (CV) of stride time and velocity. Stable gait has been linked to longer stride length, shorter stride time, longer swing time, faster velocity, and lower variability [18–21].

### 2.5. Statistical Analyses

For BAI, HR, and BP (i.e., our measures of anxiety), we performed analogous paired *t*-tests for the alprazolam and placebo sessions separately, contrasting measures of anxiety obtained at the baseline relative to those obtained at the final block of each session. Furthermore, we contrasted our anxiety measures obtained in the alprazolam versus placebo sessions at the (a) baseline block and (b) final block. These contrasts were planned to investigate whether alprazolam had its intended effect of reducing anxiety.

For gait and gait variability measures, we ran 2 × 4 repeated measures (RM) analyses of variance (ANOVAs), with medication (alprazolam vs. placebo) and condition (Baseline, LA, HA-Pit, and HA-Plank) as within-subject variables. In cases of sphericity violation, the Greenhouse–Geisser correction was implemented. Simple effects were investigated using Tukey's honest significant difference (HSD) post-hoc *t*-tests for any significant main or interaction effects.

## 3. Results

### 3.1. Health and Demographics

Health, demographic, and questionnaire measures are presented in Table 1. According to the NFOG questionnaire, patients were mild-to-moderate freezers with scores ranging from 4 to 14 (Table 1). Patients did not suffer from cognitive impairment (i.e., scores >26 on the MoCA) [14], which avoids any potential confounding effects of cognitive impairment related to FOG, gait performance, anxiety, and anxiety-reducing medication. Only three patients were taking prescribed SSRI medication for anxiety.

### 3.2. Anxiety Measures

#### 3.2.1. Subjective Measure: BAI

In paired sample *t-*tests, we contrasted BAI scores obtained at the baseline (i.e., prior to administration of alprazolam or placebo) versus at the conclusion of the final block of gait testing for alprazolam and placebo sessions, separately. We noted a significant reduction in BAI scores from precapsule administration to the end of the final block of gait measures in the alprazolam session (*t*(9) = 2.205, *p*=0.05) but no difference in the placebo session (*t*(9) = 0.793, *p*=0.448). This provided evidence that, subjectively, anxiety was reduced by alprazolam, confirming the efficacy of our manipulation.

Paired-sample *t*-tests on BAI scores for alprazolam versus placebo sessions in the (a) baseline blocks and (b) final blocks, separately, also revealed a significant difference. At the baseline, patients on alprazolam had significantly higher anxiety scores compared to placebo (*t*(9) = 2.631, *p*=0.027). There was no significant difference comparing alprazolam and placebo sessions at the final block timepoint, however. Somewhat confirming the within-session effects (i.e., baseline versus final block), though patients on alprazolam had higher baseline anxiety scores (i.e., preadministration of alprazolam), anxiety was reported as equivalent in the final block for patients in the alprazolam and placebo sessions.

#### 3.2.2. Objective Measures: HR and Systolic BP

On HR and BP measures separately, we performed paired *t-*tests contrasting scores in the baseline versus final blocks of the alprazolam and placebo sessions, separately. No significant differences were noted for HR scores for either the alprazolam (*t*(9) = 1.59, *p*=0.14) or the placebo sessions (*t*(9) = −1.09, *p*=0.30). In contrast, systolic BP was significantly reduced following the administration of alprazolam [*t*(9) = 2.420, *p*=0.03, a difference that was not significant on placebo (*t*(9) = −1.20, *p*=0.25), suggesting anxiety reduction related to alprazolam.

Paired-sample *t*-tests were also performed comparing HR or BP for alprazolam versus placebo sessions in the (a) baseline blocks and (b) final blocks. At the baseline, HR and BP were not significantly different for alprazolam and placebo (*t*(9) = 0.32, *p*=0.75 for HR; *t*(9) = 1.49, *p*=0.17 for BP). However, in the final blocks, HR was significantly lower in the alprazolam session compared to the placebo session (*t*(9) = −2.87, *p*=0.01).

### 3.3. Gait Measures

#### 3.3.1. FOG Episodes

No main effect of medication on FOG frequency was observed (*F*(1, 9) = 0.11, MSe = 7.03, *p*=0.74). FOG episodes occurred only during the HA trials, with none arising in the LA scenarios or at the baseline. This produced a significant main effect of condition (*F*(2, 18) = 8.82, MSe = 4.53, *p* < 0.01). There was no significant interaction between medication and condition (*F*(2, 18) = 0.11, MSe = 7.03, *p*=0.89). During the HA scenarios, however, patients experienced 21 FOG episodes on alprazolam compared to 28 episodes on placebo. Assessing presence vs. absence of FOG across sessions using an exact binomial probability analysis, there was a marginally significant difference (*p*=0.05), with three patients experiencing FOG in the alprazolam session compared to six in the placebo session.

#### 3.3.2. Swing Time

**Figure 2:**
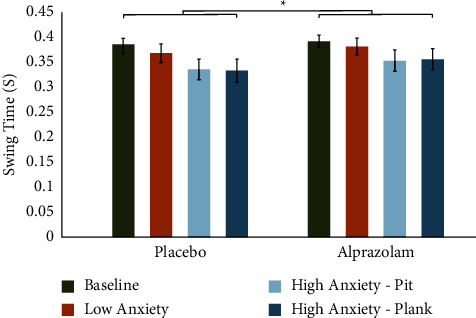
Alprazolam increases swing time during anxiety-inducing walking scenarios, compared to placebo. There were significant main effects of condition and medication. Paired sample *t*-tests demonstrate a significant increase in swing time while on alprazolam for both HA and LA conditions (HA-plank: *t*(9) = 2.79, *p*=0.02; HA-pit: *t*(9) = 3.07, *p*=0.01; and LA: *t*(9) = 2.45, *p*=0.03). Swing time was similar at the baseline for both medication states (*t*(9) = 1.08, *p*=0.30). Error bars represent the standard error of the mean (SEM).

Our 2 × 4 ANOVA with medication (alprazolam vs. placebo) and condition (baseline, LA, HA-pit, and HA-plank) as within-subject variables showed a significant main effect of medication (*F*(1, 9) = 12.57, MSe = 0.0004, *p*=0.006), in which alprazolam administration led to prolongation of swing time in all three VR gait conditions (Figure 2). We also found a significant main effect of condition (*F*(3, 27) = 11.79, MSe = 0.0008, *p*=0.005; Greenhouse–Geisser corrected), in which patients demonstrated a reduction in swing time in the HA conditions compared to the LA condition. The medication × condition interaction was not significant (*F*(3, 27) = 1.83, MSe = 0.0001, *p*=0.15).

Other direct gait variables, such as step length, step time, and cadence, were not significantly affected by alprazolam (Table 2).

### 3.4. Gait Variability

#### 3.4.1. Stride Time Variability

**Figure 3:**
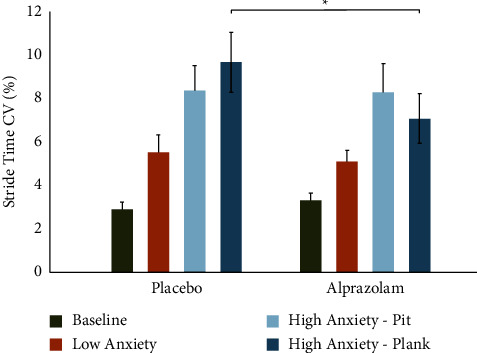
Alprazolam reduces stride time variability while walking in an anxiety-inducing virtual environment. No significant main effect of medication was observed, but there was a significant main effect of condition (*p* < 0.001) and condition × medication interaction (*p*=0.02). Paired samples *t*-test between HA-plank conditions showed significant reduction of stride time variability on alprazolam compared to placebo (*t* = −2.72, *p*=0.02). Error bars represent the standard error of the mean (SEM).

There was no main effect of medication on stride time-CV (*F*(1, 9) = 1.41, MSe = 6.38, *p*=0.26). However, higher anxiety scenarios increased stride time-CV, evidenced by a main effect of condition (*F*(3, 27) = 19.19, MSe = 6.80, *p* < 0.001, Greenhouse–Geisser corrected). Furthermore, there was a medication × condition interaction (*F*(3, 27) = 3.87, MSe = 2.26, *p*=0.03, Greenhouse–Geisser corrected). In Tukey's post-hoc tests, we found that alprazolam significantly reduced stride time-CV in the HA-plank condition (*t*(9) = −2.72, *p*=0.02), but this effect was not seen on the LA or HA-pit conditions (Figure 3).

#### 3.4.2. Stride Velocity Variability

**Figure 4:**
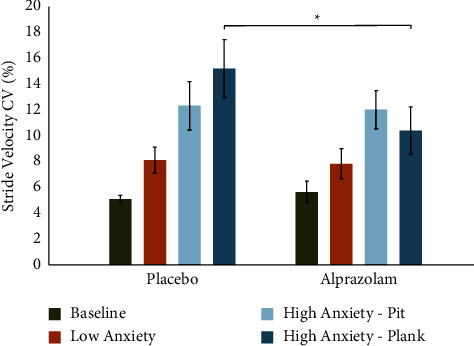
Alprazolam reduces stride velocity variability while walking in an anxiety-inducing virtual environment. There was a significant main effect of condition (*p* < 0.001) and a condition × medication interaction (*p*=0.01). A paired samples *t*-test between HA-plank conditions showed significant reduction of stride velocity variability on alprazolam compared to placebo (*t* = −2.39, *p*=0.04). Error bars represent the standard error of the mean (SEM).

There was no main effect of medication (*F*(1, 9) = 1.268, MSe = 22.96, *p*=0.289). The main effect of condition (*F*(3, 27) = 26.369, MSe = 9.46, *p* < 0.001, Greenhouse–Geisser corrected) was found showing a significant increase in stride velocity-CV in the HA conditions. We again found a medication × condition interaction (*F*(3, 27) = 4.025, MSe = 29.38, *p*=0.034, Greenhouse–Geisser corrected). Post-hoc tests revealed a significant reduction in stride velocity-CV following the administration of alprazolam, specifically in the HA-plank condition (*t*(9) = −2.391, *p*=0.040), but not in the HA-pit or in the LA conditions (Figure 4).

## 4. Discussion

In the current study, we evaluated whether anxiety plays an etiological role in FOG, comparing conditions that were otherwise identical in their sensory complexity, as well as their physical and motor demands. We altered the level of anxiety by (a) creating VR scenarios that varied in perceived elevation of the plank on which patients walked and (b) providing an anxiolytic (i.e., alprazolam) versus a placebo, while measuring episodes of FOG and other gait parameters using a GAITRite mat. Our pharmacological manipulation was performed in a double-blind manner, and all our measures were collected within-subject, with medication session (i.e., alprazolam vs. placebo) counterbalanced across participants.

In cognitively intact PD patients who had a history of FOG, we confirmed that elevated-plank VR scenarios increased (a) subjective and objective measures of anxiety and (b) the prevalence of FOG, while worsening (c) swing time and (d) gait variability, making gait more unstable overall. FOG appeared only in HA conditions in this study, supporting the contention that anxiety provokes episodes of freezing in PD patients with FOG. Swing time was significantly reduced in all virtual conditions (i.e., LA, HA-pit, and HA-plank) compared to the baseline (i.e., ambulation on GAITRite mat without VR goggles), indicating that patients were spending less time with one foot midair, denoting a less secure gait. Finally, higher anxiety scenarios increased variability of stride time and stride velocity, which produces a more poorly balanced gait. Taken together, these results demonstrate that increasing anxiety alone, in conditions that were otherwise matched in sensory, cognitive, and motor demands, evoked FOG and worsened gait parameters related to stability and FOG. This pattern of results replicates those of Ehgoetz Martens and colleagues [5], who developed this paradigm.

In addition to replicating the findings of Ehgoetz Martens and colleagues [5], we further investigated whether we could pharmacologically alleviate anxiety and, in turn, reduce episodes of FOG and improve FOG-related gait abnormalities. Indeed, administering low-dose alprazolam in PD patients with FOG reduced subjective (i.e., modified BAI scores) and objective (i.e., HR and BP) measures of anxiety, whereas introducing an identical placebo capsule did not. These results demonstrate that a single, low dose of alprazolam produces a clear anxiolytic effect in PD patients with FOG and correspondingly decreases the number of patients who experienced FOG compared to placebo (*p*=0.05). Alprazolam also had positive effects on gait parameters that are associated with FOG by increasing swing time and decreasing gait variability [22, 23].

Longer swing times translate to more stable gait [21, 24, 25], whereas reduced swing times are associated with greater walking instability, falls, and incapacitation [24]. PD patients usually have reduced swing time compared to their healthy peers [21]. Alprazolam reduced variability in both stride time and stride velocity but only in the HA-plank condition. Previous studies have shown that increased gait variability, including in stride time [26] and stride length [27–29] correlate with FOG, gait instability, and risk of falling. Taken together, the ameliorating effect of alprazolam on FOG and gait was demonstrated.

Previous studies have shown that reduced swing time [21, 26] and cadence [21], as well as increased gait variability, including stride time CV [26], stride length CV [27–29], and step time [30], correlate with FOG, gait instability, and risk of falling. Alprazolam improved swing time, stride time CV, and stride length CV but had no significant effect on other gait measures, such as cadence, step length, and step time. Theoretically, improving any of these gait parameters could lead to reductions in FOG through improved gait stability.

FOG is a symptom in PD that greatly impacts quality of life and threatens independence. Over the past decade, the association between anxiety and FOG has become clear [5, 7, 31–33]. Apart from the findings of Ehgoetz Martens et al. [5], most previous investigations of anxiety and FOG in PD have been correlational. We have demonstrated that inducing anxiety increases the prevalence of FOG and worsens gait parameters associated with FOG, whereas reducing anxiety through pharmacological means decreases the frequency of FOG episodes and improves swing time, as well as variability of stride time and stride velocity. As well as demonstrating that modulation of anxiety itself directly impacts FOG and gait, this study raises the possibility of new strategies for the treatment of FOG, a particularly intractable symptom leading to falls, and ultimately the loss of independent ambulation and function.

### 4.1. Limitations

In the current study, we used a benzodiazepine with rapid anxiolytic effects to promptly reduce anxiety, allowing us to assess the effect on FOG and other components of gait immediately, during the session. In the long term, benzodiazepines are not the preferred treatment for anxiety in PD patients due to their effects on cognition and alertness, as well as the potential for dependence and abuse. This study aimed to prove the principle that anxiety reduction itself can ameliorate FOG and associated gait abnormalities in PD patients with FOG.

A small number of PD patients participated in the current study. Recruiting patients for this study was very challenging. To optimize safety and tolerability of the VR methodology, as well as of our alprazolam manipulation, our participant selection criteria were highly constrained. In this first investigation, we included only PD patients (a) with FOG, (b) who could safely ambulate without an assistive device for 10 meters, while wearing VR goggles, and (c) who had normal cognition. FOG generally arises at later stages in PD, coincident with (a) worsening of other gait parameters that frequently require the use of an assistive device and (b) cognitive impairment. Despite our low sample size, potentially due to the advantages of our within-subject design, we found predicted effects of our manipulations on subjective and objective measures of anxiety, prevalence of FOG, and other FOG- and stability-related gait parameters. Though this small sample potentially limits the generalizability of our results, in this first investigation, safety was our highest priority.

Having demonstrated that anxiety alleviation improves FOG in a small sample of PD patients, further investigations seem warranted. These positive findings could motivate trials in larger samples with anxiety-reduction goals achieved using better-tolerated anxiolytics, such as SSRIs, and measuring effects on gait in the lab, as well as in more naturalistic settings. Behavioural approaches to anxiety mitigation, such as cognitive-behavioural therapy, have proven effective in PD [34, 35], with preliminary efficacy in reducing the fear of falling [36]. Though limited in their efficacy, there are a number of nonpharmacological techniques aimed at reducing FOG, such as cueing, as well as cognitive, obstacle, and dual-task training [37, 38]. Future studies could investigate the effect on FOG of SSRI optimization, along with or compared to, these behavioural approaches. These future studies will also help disentangle whether our current findings with alprazolam on FOG and gait owe to anxiety mitigation per se, rather than to alprazolam's GABAergic modulation of gait pathways. The results of this study suggest potential new therapeutic avenues for treating FOG. SSRIs and behavioural approaches to anxiety and FOG alleviation are safely used in PD already, though standardized treatment regimens, titrated to anxiety and gait endpoints have not previously been explored. Our findings suggest novel pathways for treating FOG, an intractable symptom that greatly threatens independence and quality of life in PD.

## 5. Conclusion

In conclusion, we have shown that anxiety-provoking VR scenarios increase the prevalence of FOG and worsen gait parameters related to stability and FOG. Complementing these findings, a fast-acting benzodiazepine, alprazolam, reduced FOG and improved those gait parameters that were worsened by anxiety-producing VR scenarios. This study suggests that anxiety is a causal factor in FOG that might impact other aspects of gait stability in PD. To our knowledge, this is the first study demonstrating the ameliorating effect of anxiolytics on FOG, potentially opening an entirely new therapeutic avenue for addressing this relatively intractable symptom that threatens independence and quality of life. This study will motivate future research into the impacts of better-tolerated anxiolytics on FOG and gait. Exploring the effects on FOG of anxiolytics such as SSRIs that impact non-GABAergic pathways will extend and clarify the current findings.

## Figures and Tables

**Table 1:** Health and demographic information for Parkinson's disease patients with freezing of gait.

	Mean (±SD)
Age (years)	68.7 (1.97)
Disease duration (years)	6.4 (4.68)
NFOG	10 (3.36)
MoCA	27.50 (1.50)
LEDD	886 (385.5)
Oxford happiness	4.25 (0.61)
Verbal fluency	
F	13.30 (4.29)
A	12.10 (5.36)
S	14.70 (6.14)
Animals	19.90 (4.81)
ANART	124.56 (5.62)
Sleepiness	8.80 (5.54)

Results are reported as means (±SD). NFOG = new freezing of gait questionnaire; MoCA = Montreal cognitive assessment; LEDD = levodopa equivalent daily dose; ANART = American national adult reading test. LEDD = L-dopa dose + (L-dopa dose × 1/3) if on entacapone + (bromocriptine (mg) × 10) + (cabergoline or pramipexole (mg) × 67) + (ropinirole (mg) × 20) + (pergolide (mg) × 100) + (apomorphine (mg) × 8).

**Table 2:** Repeated measures 2 × 4 ANOVA for gait variables.

Variable name	Comparison	*F*	*df*	*p*
Step length	Main effect of medication	0.03	1	0.85
	Main effect of condition	33.83	3	<0.001
	Medication vs condition interaction	2.12	3	0.16

Step time	Main effect of medication	2.72	1	0.13
	Main effect of condition	1.58	3	0.21
	Medication vs condition interaction	2.48	3	0.09

Cadence	Main effect of medication	3.59	1	0.09
	Main effect of condition	1.05	3	0.38
	Medication vs condition interaction	1.16	3	0.34

Main effect of medication (alprazolam vs. placebo), condition (baseline, LA, HA-pit, and HA-plank), and interactions are included.

## Data Availability

Requests for access to the data should be made to the corresponding author.

## Additional Points

*Key Messages.* freezing of gait (FOG) is a debilitating symptom that has few treatment possibilities. This symptom is one of the main risk factors for falls and worsening of quality of life in patients with Parkinson's disease (PD). FOG has been closely linked to anxiety. Here, we investigated the effect of acutely increasing or decreasing anxiety on FOG and related gait measures. We confirmed a clear relation between anxiety and FOG, where freezing and gait impairment were significantly impacted by high anxiety conditions. In contrast, introducing a fast-acting anxiolytic (i.e., alprazolam) significantly improved subjective and objective measures of anxiety, as well as gait parameters related to freezing, concomitantly. We showed that a low dose of alprazolam, given once, resulted in clear improvement in FOG and gait stability. These findings could motivate investigations of anxiolytics that are better tolerated for a long term, such as serotonin reuptake inhibitors, titrated to gait symptoms as a potential therapy of FOG.
